# Emergency Physician-performed Echocardiogram in Non-ST Elevation Acute Coronary Syndrome Patients Requiring Coronary Intervention

**DOI:** 10.5811/westjem.60508

**Published:** 2023-12-22

**Authors:** Ting Xu Tan, Donald Wright, Cristiana Baloescu, Seohyuk Lee, Christopher L. Moore

**Affiliations:** * Yale University School of Medicine, Department of Emergency Medicine, New Haven, Connecticut; † St. Joseph’s Medical Center, Department of Emergency Medicine, Stockton, California; ‡ Yale University School of Medicine, New Haven, Connecticut

## Abstract

**Introduction:**

Identification of patients not meeting catheterization laboratory activation criteria by electrocardiogram (ECG) but who would benefit from early coronary intervention remains challenging in the emergency department (ED). The purpose of this study was to evaluate whether emergency physician (EP)-performed point-of-care transthoracic echocardiography (POC TTE) could help identify patients who required coronary intervention within this population.

**Methods:**

This was a retrospective observational cohort study of adult patients who presented to two EDs between 2018–2020. Patients were included if they received a POC TTE and underwent diagnostic coronary angiography within 72 hours of ED presentation. We excluded patients meeting catheterization laboratory activation criteria on initial ED ECG. Ultrasound studies were independently reviewed for presence of regional wall motion abnormalities (RWMA) by two blinded ultrasound fellowship-trained EPs. We then calculated test characteristics for coronary intervention.

**Results:**

Of the 221 patient encounters meeting inclusion criteria, 104 (47%) received coronary intervention or coronary artery bypass grafting (CABG) referral. Overall prevalence of RWMA on POC TTE was 35% (95% confidence interval [CI] 29–42%). Presence of RWMA had 38% (95% CI 29–49%) sensitivity and 68% (95% CI 58–76%) specificity for coronary intervention/CABG referral. Presence of “new” RWMA (presence on EP-performed POC TTE and prior normal echocardiogram) had 43% (95% CI 10–82%) sensitivity and 93% (95% CI 66–100%) specificity for coronary intervention/CABG referral. The EP-performed POC TTE interpretation of RWMA had 57% (95% CI 47–67%) sensitivity and 96% (95% CI 87–100%) specificity for presence of RWMA on subsequent cardiology echocardiogram during the same admission.

**Conclusion:**

Presence of RWMA on EP-performed POC TTE had limited sensitivity or specificity for coronary intervention or referral to CABG. The observed specificity appeared to trend higher in subjects with a prior echocardiogram demonstrating absence of RWMA, although a larger sample size will be required to confirm this finding. The EP-performed POC TTE RWMA had high specificity for presence of RWMA on subsequent cardiology echocardiogram. Further evaluation of the diagnostic performance of new RWMA on EP-performed POC TTE with a dedicated cohort is warranted.

Population Health Research Capsule What do we already know about this issue?

*Presence of regional wall motion abnormalities (RWMA) can indicate cardiac ischemia and may predict occlusive disease in non-ST elevation acute coronary syndrome (NSTE-ACS) patients.*

What was the research question?

*Are RWMAs associated with coronary intervention in NSTE-ACS patients?*

What was the major finding of the study?

*RWMA had 38% (95% CI 29–49%) sensitivity and 68% (95% CI 58–76%) specificity for coronary intervention or surgical referral.*

How does this improve population health?

*Understanding the diagnostic test performance of RWMA in NSTE-ACS has the prospect to improve use of early angiography for high-risk individuals.*

## INTRODUCTION

Every year, an estimated 600,000 patients present to the emergency department (ED) with acute myocardial infarction (AMI).^1^ Acute myocardial infarctions are historically divided into ST-elevation acute coronary syndrome (STE-ACS) and non-ST-elevation acute coronary syndrome (NSTE-ACS) based on electrocardiogram (ECG) findings of STE in regional leads. In patients with STE-ACS, guidelines recommend immediate coronary angiography and revascularization (Class I recommendation).^2^ However, in patients presenting with symptoms concerning for ACS without classic STE on their initial ED ECG, ED workup aimed at identifying patients likely to benefit from coronary intervention remains challenging. The current American Heart Association (AHA) guidelines for patients suspected to have NSTE-ACS recommend serial ECGs and cardiac troponins every 3–6 hours (both Class I recommendations).^3^ These approaches are time consuming and could delay coronary intervention, as well as impact downstream morbidity and mortality.^4^

Approximately 25% of NSTE-ACS patients have been subsequently found to have occlusive coronary disease.^4^ This population fares poorly, with larger infarcts, higher cardiac biomarkers, and greater mortality than those without obstructive disease.^5^ ^,^ ^6^ Among studies that evaluated patients with high-risk NSTE-ACS, such as the RIDDLE NSTEMI and Sisca trials, early reperfusion was associated with reduced risk of death or new MI and cumulative incidence of death, MI, or urgent revascularization at 30 days, respectively.^7^ ^,^ ^8^ For these reasons, higher risk patients with findings in the ED concerning for an occlusive coronary process are likely to benefit from expedited intervention.

Prior studies of cardiology-performed transthoracic echocardiography (cardiology TTE) have shown that in patients presenting with symptoms concerning for ACS, RWMA appeared earlier than ECG changes and were more sensitive for AMI and critical coronary stenosis.^9^ ^–^ ^12^ Much of the prior research has focused on using RWMA to identify patients with AMI and predict in-hospital complications and long-term cardiac events.^9^ ^–^ ^11^ ^,^ ^13^ ^–^ ^15^ While several studies have highlighted the rates of revascularization and acute coronary occlusion in their patient population, these were done by non-emergency physicians (EP) or in a non-ED setting.^9^ ^,^ ^12^ ^–^ ^14^ ^,^ ^16^ ^,^ ^17^ In addition, many of these studies focused on the capability of RWMA to detect AMIs (as determined by cardiac biomarkers, clinical symptoms, and/or ECG changes) instead of the identification of patients likely to receive intervention based on the presence of acute coronary occlusion as evidenced by cardiac catheterization.^9^ ^–^ ^11^ ^,^ ^13^ ^,^ ^14^ ^,^ ^16^ ^,^ ^17^

In ED patients without STE on initial ECG, EP-performed point-of-care transthoracic echocardiography (POC TTE) may help identify patients who have an intervenable coronary lesion but have been incompletely investigated. This is supported by a prior small case series, which showed that detection of RWMA by EP-performed POC TTE correlated with the vessel territories on subsequent intervention.^18^ In this study, our primary objective was to describe the diagnostic test characteristics of RWMA found on EP-performed POC TTE for percutaneous coronary intervention (PCI) or referral for coronary artery bypass grafting (CABG) among NSTE-ACS patients. The secondary objective was to perform subgroup test characteristic analysis based on troponin elevation and prior cardiology TTE without RWMA.

## METHODS

### Study Design and Setting

This was a retrospective observational cohort study conducted at an urban academic hospital system that includes a quaternary-care academic center ED with 80,600 annual visits and a freestanding suburban ED with 24,600 annual visits. The academic center has 24-hour catheterization laboratories and in-house interventional cardiology. Patients requiring cardiology consultation or admission in the freestanding ED are transferred to the academic center for further care. The study was approved by the institutional review board prior to commencement.

### Participants

Patients who presented to the EDs between 2018–2020 were included if they were 1) >18 years old; 2) received an EP-performed POC TTE during the visit; and 3) underwent diagnostic cardiac catheterization for suspected ACS within 72 hours of ED presentation. Patients were excluded if they had an initial ED ECG that met catheterization laboratory activation criteria or if inadequate images were obtained to evaluate RWMA on EP-performed POC TTE.

### Outcome Variables

The primary outcomes of the study were diagnostic test characteristics (sensitivity, specificity, positive and negative likelihood ratios) of RWMA identified on EP-performed POC TTE for coronary intervention or referral for coronary artery bypass grafting (CABG) in patients undergoing coronary angiography. A priori subgroups were designated based on troponin elevation and prior absence of RWMA on cardiology TTE.

### Data Sources/Measurement

Investigators included three emergency ultrasound fellowship-trained EPs, two emergency medicine residents, and one medical student. All attending/resident investigators received emergency ultrasound training during residency and/or fellowship.

Patient data was deidentified, given a unique study ID, and stored in a HIPAA-compliant cloud-based storage application (Box, Inc., Redwood City, CA). Procedure reports and inpatient notes were reviewed to identify patients who underwent PCI or were referred for CABG within 24 hours of coronary angiography as a composite outcome. Prior cardiology TTE reports were also coded for existing RWMA. Chart abstraction was performed by a resident author who was blinded to EP-performed POC TTE result using a standardized abstraction form with identical fields for each encounter.

The EP-performed POC TTE studies were obtained from Qpath (Telexy Healthcare, Maple Ridge, British Columbia, Canada), the storage and workflow manager where all studies obtained in the ED are stored. Initial studies were collected in the usual course of clinical care by clinicians and learners involved in the care of the patient with common indications including evaluation for pericardial effusion, left ventricular function, and right ventricular dilation. Of note, dedicated assessment of regional walls is not a routine part of our institutional EP-performed POC TTE protocol.

RWMA on EP-performed POC TTE was independently assessed by two ultrasound fellowship-trained attendings, who were blinded to the other’s initial interpretation and to all chart/outcome data. Interpretation of these parameters was based on global qualitative assessment as “present,” “absent,” or “uninterpretable” due to insufficient images obtained. Uninterpretable ultrasounds were excluded. An a priori adjudication plan was in place to evaluate any cases in which the two reviewers disagreed on the interpretability of an ultrasound or presence of RWMA. In these cases, the studies were jointly reviewed and discussed until a final interpretation was identified.

All ECGs obtained in the ED during the visit were screened by a senior resident author who was blinded to other chart data and EP-performed POC TTE result. The interpretations were performed based on AHA guidelines for STEMI and the modified Sgarbossa’s criteria.^2^ ^,^ ^19^ Patients with any ED ECG potentially meeting STEMI criteria had their initial ED ECG independently interpreted by the two attending authors to assess whether they would activate the catheterization laboratory based on the ECG, assuming there were symptoms consistent with acute coronary syndrome. Any disagreements were reviewed by a third attending EP, who provided the final adjudication. We excluded patients with an initial ED ECG who were felt to meet catheterization laboratory criteria.

Both troponin I (Abbott Laboratories, Chicago, IL) and troponin T (Roche Diagnostics, Basel, Switzerland) were available in the ED for this study population. We included all troponin values obtained while the patient was in the ED. Institutional laboratory threshold values were used to delineate whether a troponin was positive. A positive troponin I value was >0.08 nanograms per milliliter (ng/mL). A positive troponin T value was >0.01 ng/mL.

We performed a planned subgroup analysis on subjects with a potentially “new” RWMA, defined as those with a prior echocardiogram on file in which the most recent report explicitly commented on the absence of any RWMA.

### Statistical Methods

Based on prior unpublished pilot data, power analysis suggested that to minimize the 95% confidence interval [CI] to a width of 20% or less for sensitivity of RWMA in patients receiving coronary intervention/referral, 104 subjects would be required in each outcome group.

We conducted data analysis in R (R Foundation for Statistical Computing, Vienna, Austria) using the RStudio interface (RStudio Inc., Boston, MA). Demographic characteristics were tabulated by whether subjects received a coronary intervention and/or referral to CABG with differences evaluated by the *t*-test for continuous variables and chi-square test for categorical variables. We calculated diagnostic performance characteristics with the epiR package.

## RESULTS

**Figure 1.**
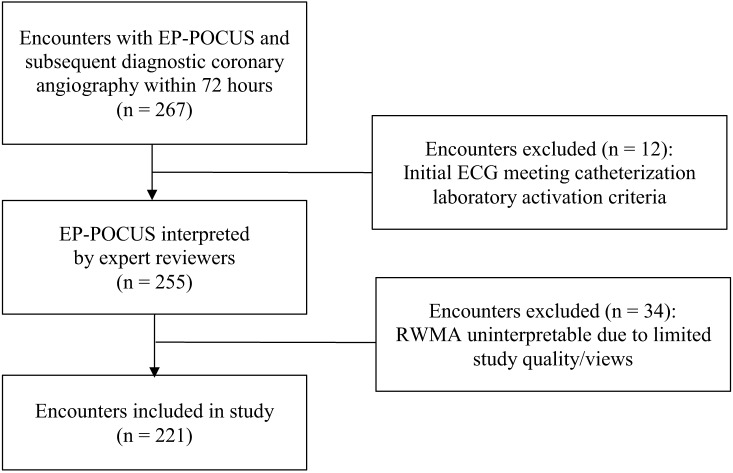
Flow diagram of screened and included patient encounters among subjects evaluated with emergency physician-performed point-of-care echocardiography (during assessment for acute coronary syndrome). *EP*, emergency physician; *POC TTE*, point-of-care transthoracic echocardiogram; *ECG*, electrocardiogram; *RWMA*, regional wall motion abnormality.

A total of 221 patient encounters were included in the study, of whom 104 (47%) received a coronary intervention and/or referral to CABG (Figure 1). Mean age of subjects was 64.8 years, and 33.9% of the sample was female (Table 1). The coronary intervention/referral group was statistically older (mean age 67.1), more likely to be male (78.8%), and less likely to identify as Black.

**Table 1.** Demographics of participants by coronary intervention status.

Patient characteristics	No coronary intervention (n = 117)	Coronary intervention (n = 104)	Total (n = 221)	*P*-value
RWMA present on EP-POC TTE, no. (%)	38 (32.5%)	40 (38 5%)	78 (35.3%)	0.62
ED setting, no. (%)				
Quaternary care academic ED	104 (88.9%)	90 (86.5%)	194 (87.8%)	0.74
Suburban freestanding ED	13 (11.1%)	14 (13.5%)	27 (12.2%)	
Age, mean, yr (SD)	62.8 (14.1)	67.1 (12.1)	64.8 (13.4)	0.01^*^
Female, no. (%)				
Female	53 (45.3%)	22 (21.2%)	75 (33.9%)	<0.01^*^
Male	64 (54.7%)	82 (78.8%)	146 (66.1%)	
Ethnicity, no. (%)				
Hispanic or Latino	13 (11.1%)	9 (8.7%)	22 (10.0%)	0.83
Non-Hispanic	103 (88.0%)	94 (90.4%)	197 (89.1%)	
Other/unknown	1 (0.9%)	1 (1.0%)	2 (0.9 %)	
Race, no. (%)				
Asian	3 (2.6%)	3 (2.9%)	6 (2.7%)	0.04^*^
Black	23 (19.7%)	7 (6.7%)	30 (13.6%)	
White	81 (69.2%)	86 (82.7%)	167 (75.6%)	
Other/unknown	10 (8.5%)	8 (7.7%)	18 (8.1%)	

*RWMA*, regional wall motion abnormality; *POC TTE*, point-of-care transthoracic echocardiogram.

In our overall study population, RWMA was present in 35% (95% CI 29–42%) of cases. Interrater reliability for presence of RWMA prior to adjudication was 0.37 (95% CI 0.25–0.49). Presence of RWMA had 38% (95% CI 29–49%) sensitivity and 68% (95% CI 58–76%) specificity for coronary intervention/referral. The positive likelihood ratio was 1.18 (95% CI 0.83–1.69) and negative likelihood ratio was 0.91 (95% CI 0.75–1.11). Prevalence of RWMA and test characteristics for intervention were similar in the subgroup of patients with an elevated troponin at any time in the ED. Among these cases, RWMA was present in 37% (95% CI 30–45%). Presence of RWMA had 41% (95% CI 30–51%) sensitivity and 66% (95% CI 56–75%) specificity for coronary intervention/referral. The positive likelihood ratio was 1.18 (95% CI 0.82–1.71) and negative likelihood ratio was 0.90 (95% CI 0.72–1.13).

We identified a small subgroup of 21 encounters with the most recent cardiology TTE explicitly documenting the absence of any RWMA. In this subgroup, RWMA was now present in 19% (95% CI 5–42%). Presence of RWMA had 43% (95% CI 10–82%) sensitivity and 93% (95% CI 66–100%) specificity for coronary intervention/referral. The positive likelihood ratio was 6.00 (95% CI 0.75–47.71) and negative likelihood ratio was 0.62 (95% CI 0.32–1.19). A summary of all test characteristics is shown in Table 2 for each group.

**Table 2.** Diagnostic test characteristics of presence of regional wall motion abnormality on emergency physician-performed point-of-care transthoracic echocardiography for coronary intervention.

Group	Prevalence, % (95% CI)	Sensitivity, % (95% CI)	Specificity, % (95% CI)	Positive likelihood ratio (95% CI)	Negative likelihood ratio (95% CI)
All encounters n = 221	35% (29–42%)	38% (29–49%)	68% (58–76%)	1.18 (0.83–1.69)	0.91 (0.75–1.11)
Elevated troponin n = 193	37% (30–45%)	41% (30–51%)	66% (56–75%)	1.18 (0.82–1.71)	0.90 (0.72–1.13)
No prior RWMA n = 21	19% (5–42%)	43% (10–82%)	93% (66–100%)	6.00 (0.75–47.71)	0.62 (0.32–1.19)

*CI*, confidence interval; *RWMA*, regional wall motion abnormality; *CI*, confidence interval.

In 158 encounters (71.5%), a subsequent cardiology TTE was identified, which explicitly commented on the presence or absence of RWMA during the same admission. The ED RWMA had 57% (95% CI 47–67%) sensitivity and 97% (95% CI 87–100%) specificity for RWMA on cardiology TTE. There were only two cases in which an RWMA was felt to be present in the ED but not on cardiology TTE.

## DISCUSSION

The determination of which ED NSTE-ACS patients would benefit from expedited coronary intervention has been a longstanding challenge. The traditional paradigm of dividing patients into STE-ACS vs NSTE-ACS groups resulted in about 25% of missed acute coronary occlusion that would have been potentially amenable to more urgent intervention.^4^ More recently, a new paradigm of stratifying patients into occlusion myocardial infarction (OMI) vs non-occlusion myocardial infarction (NOMI) has been proposed.^20^ This paradigm is similar to the distinction made in the 2022 American College of Cardiology Expert Consensus Decision pathway on the Evaluation of Acute Chest Pain in the Emergency Department, which makes a distinction in management recommendations between NSTE-ACS Type 1 (occlusive disease related to atherosclerotic plaque rupture and thrombosis) and Type 2 (non-occlusive process related to an oxygen supply/demand mismatch).^21^ Our study adds to this body of work by demonstrating the potential role for detection of RWMA on EP-performed POC TTE as part of a comprehensive evaluation for suspected occlusive coronary disease.

To our knowledge, this is the first large study evaluating the test characteristics of RWMA on EP-performed POC TTE for coronary intervention or referral to CABG. Our study demonstrated a relatively modest 68% specificity of RWMA on EP-performed POC TTE for intervention/referral. This finding is lower although comparable to earlier studies showing RWMA in cardiology-performed TTE to be 78% specific for significant coronary artery disease (CAD) in the NSTE myocardial infarction (NSTEMI) population.^14^ The 35% prevalence of RWMA in our NSTEMI population was comparable to rates reported by Ha et al in their review.^22^ Our reported sensitivity of RWMA for coronary intervention/CABG referral in patients with elevated troponins does appear to be lower than previously reported.

Prior studies noted RWMA on cardiology TTE in the ED in 77–92% of NSTEMI patients.^13^ ^,^ ^14^ ^,^ ^17^ In the prehospital setting, Bergmann et al found that prehospital RWMA on EP-performed POC TTE was higher at 91% sensitivity for NSTEMI and correlated to occluded coronary vessels seen in 85% of PCI cases.^17^ Peels et al found RWMA to be 88% sensitive in significant CAD.^14^ Differences in test characteristics may be partially attributable to inclusion of subjects with prior known CAD, history of MI, congestive heart failure, or prior RWMA, when other studies excluded these patient populations.^12^ ^,^ ^14^ ^,^ ^17^

Another likely factor contributory to our lower sensitivity is the inclusion of EP-performed POC TTE for indications other than assessment of RWMA, as dedicated assessment of regional walls is not part of our typical institutional EP-performed POC TTE protocol. These studies were primarily conducted for the assessment of alternative non-ACS entities in the clinical differential and, thereby, may have inadequately visualized some regional walls. Images were also obtained by a variety of individuals, including attending physicians, resident physicians, other clinicians, and learners. The combination of diverse indications for studies and variable experience of those performing sonography likely also contributed to the limited interrater reliability on subsequent expert interpretation due to variable image quality and the high rate of studies felt to be uninterpretable due to insufficient views obtained. In retrospect, given the lower than expected interrater reliability, future studies should consider more robust adjudication schemes such as a third rater and prescribed protocols for image acquisition to ensure adequate study quality.

It should also be noted that presence of a positive ED troponin did not significantly impact our EP-performed POC TTE test characteristics. This is likely related to the observation that most patients in the study had an elevated troponin at some time during their ED stay.

The most interesting subgroup in this study consisted of encounters with “new” RWMA on EP-performed POC TTE. While findings in this population were limited by sample size, the likelihood ratio and specificity appeared to trend higher than the overall study population. This suggests they may be a promising population for further ED-based studies, particularly as electronic health record integration progresses, and higher numbers of patients have accessible prior cardiology TTE reports. The presence of a new RWMA in a patient with a previously normal echocardiogram does seem reasonably likely to represent an occlusive coronary process given the physiology of RWMA development, although the time course of this event likely depends on the age of the prior echocardiogram.

While our results yielded a mildly positive point estimate for the positive likelihood ratio of any RWMA in predicting need for intervention, the width of the CIs prevents us from making any definitive conclusions about newly identified RWMA. It is feasible that a larger sample size, or one that is prospectively collected and intended to focus on RWMA, could be more definitive. As old myocardial scarring may cause RWMA, it makes sense that not all RWMA are indicative of acute occlusion, and we were hampered in determining the presence of “new” RWMA by the relative paucity of prior normal TTEs.

Finally, we found EP-performed POC TTE interpretation of RWMA to be 96% specific to cardiology detection of RWMA. This finding is comparable to Saglam et al who found a specificity of 92% when comparing EP and cardiology TTE interpretation.^23^ Overall, these results suggest that RWMAs diagnosed by EPs are persistent on subsequent echocardiography. The lower sensitivity may partially relate to temporal effects, as cardiology TTEs were performed later during the admission after which further ischemia may have led to the development of an RWMA not present at the time of ED assessment.

## LIMITATIONS

This study was conducted at an academic quaternary-care system with an active emergency ultrasound training program, which may limit the generalizability of the results to institutions with less expertise in point-of-care ultrasound. However, ultrasound training has become widespread throughout EM residency training, and there is likely general and growing familiarity with point-of-care ultrasound in non-academic settings. Prior studies have also shown that EM attendings and residents with limited prior ultrasound experience can be effectively trained to detect RWMA abnormalities on POC TTE.^18^ ^,^ ^23^ ^–^ ^25^ Because the study population only included patients not meeting catheterization laboratory activation criteria on initial ED ECG, but who underwent coronary angiography, these patients were likely considered higher risk for intervenable occlusion by either the ED or inpatient team. Thus, our RWMA findings in EP-performed POC TTE should be interpreted with caution in patients in lower risk categories. We did not include patients who may have had OMI on coronary angiography but were medically managed as these patients could not be reliably identified with the available clinical documentation. Additionally, the specific walls involved on EP-performed POC TTE were not correlated to the involved vessels on angiography, presenting an area for further research. Finally, given the study design it was not possible to establish a causal relationship between RWMA and coronary intervention.

## CONCLUSION

Presence of regional wall motion abnormalities on emergency physician-performed point-of-care transthoracic echocardiogram had limited sensitivity or specificity for coronary intervention or referral to coronary artery bypass grafting. The observed specificity was higher in subjects with a prior echocardiogram demonstrating absence of RWMA, but the certainty of this finding was limited by our small sample size. Emergency physician-performed POC TTE RWMA had high specificity for presence of RWMA on subsequent cardiology TTE. Future studies to evaluate the test characteristics in a larger group of subjects with prior absent RWMA on cardiology TTE are needed.
